# What should be the preferred exercise modality for overweight and obese individuals? Protocol for a systematic review and network meta-analysis

**DOI:** 10.1186/s13643-019-0964-1

**Published:** 2019-02-04

**Authors:** Lene Aasdahl, Vegard Moe Iversen, Eva Skovlund, Dagfinn Aune, Marius Steiro Fimland

**Affiliations:** 1 Department of Public Health and Nursing, Faculty of Medicine and Health Sciences, Norwegian University of Science and Technology, Trondheim, Norway; 2 Unicare Helsefort Rehabilitation Centre, Rissa, Norway; 3 Department of Epidemiology and Biostatistics, School of Public Health, Imperial College London, London, UK; 4 Department of Nutrition, Bjørknes University College, Oslo, Norway; 5 Department of Endocrinology, Morbid Obesity and Preventive Medicine, Oslo University Hospital, Oslo, Norway; 6 Department of Neuromedicine and Movement Science, Faculty of Medicine and Health Sciences, Norwegian University of Science and Technology, Trondheim, Norway

**Keywords:** Weight loss, Endurance training, Resistance training, Physical activity

## Abstract

**Background:**

Obesity is a global epidemic with profound consequences for individuals and societies. Physical exercise is important to weight reduction and weight loss maintenance. However, results on what the most effective type of exercise are unclear. The aim of this systematic review is to evaluate the effects of various exercise modalities with and without caloric restriction on body composition and metabolic health outcomes in overweight and obese adults.

**Methods:**

We will perform a comprehensive literature search in PubMed, Embase (via Ovid) and CENTRAL (through the Cochrane Library). Relevant papers will be screened in two stages: first, by title and abstract and then the full text of the remaining papers. Two reviewers will screen all the studies, and any disagreements will be discussed with and resolved by a third reviewer. Data extraction and risk of bias assessment will be performed using a pre-piloted form. A network meta-analysis combining direct and indirect treatment effect estimates will be conducted if adequate data are available. The quality of the evidence will be judged using the Grading of Recommendations Assessment, Development and Evaluation (GRADE) approach.

**Discussion:**

The results of this proposed systematic review and meta-analysis will identify whether any exercise modality should be preferred for overweight and obese adults, as well as assess the quality of the evidence. This knowledge has potential importance for clinicians and patients.

**Systematic review registration:**

PROSPERO CRD42019103371

**Electronic supplementary material:**

The online version of this article (10.1186/s13643-019-0964-1) contains supplementary material, which is available to authorized users.

## Background

A third of the world’s population is overweight or obese [1]. Obesity has immense consequences for both individuals and societies, so there is an urgent need for better treatments and care-delivery strategies [2–6]. Lifestyle modification programs consisting of diet, physical activity, exercise and behaviour therapy usually are considered to be the first treatment option [7].

Despite much discussion on the relative importance of physical activity and exercise in weight loss [8–10], it is recognised that physical activity holds a role in weight reduction and weight loss maintenance [7, 11–13]. In a Cochrane review published in 2006, Shaw et al. [12] concluded that physical exercise has a small effect on weight loss, especially when combined with dietary intervention [12]. However, physical exercise can be performed in numerous ways and designed to target muscle strength (e.g. resistance training), cardiovascular fitness (e.g. aerobic exercise), flexibility or a combination of these aims [14]. Shaw et al. [12] did not discriminate among exercise modalities, making it difficult to draw firm conclusions.

In a 2009 position paper, the American College of Sports Medicine recommended more than 150 min of moderate-intensity physical activity per week to achieve modest weight loss and more time for greater weight loss [15]. It reported that physical activity increases weight loss with modest diet restrictions but not for more severe diet restrictions [15]. They found no evidence supporting that resistance training induces weight loss but some evidence that resistance training improved body composition (increased lean body mass, reduced body fat). It was also noted that some studies found superior effects from combining resistance training and aerobic exercise compared to aerobic training alone. This finding is in line with the results of a systematic review and network meta-analysis by Schwingshackl et al. [16] comparing the effects of aerobic exercise, resistance training and a combination of the two on anthropometric and metabolic outcomes. They concluded that combined exercise induces greater weight loss than resistance training alone and a larger increase in lean body mass than aerobic exercise alone [16]. They did not perform separate analyses for diet status but included interventions with a diet applied to all intervention groups. Furthermore, they reported a general risk of bias for all the included studies, but not for the individual studies [16]. Finally, they did not report an assessment of the strength of the evidence, which is recommended for systematic reviews [17, 18].

In a more recent review and meta-analysis, Clark [19] concluded that treatment should emphasise producing high metabolic stress by engaging in progressive resistance training or high-intensity aerobic exercise (> 70% of maximal heart rate). The author found that the combination of diet and exercise is more effective than diet alone at reducing fat mass while retaining fat-free mass, and the largest effect was observed for the combination of diet and resistance training [19]. However, there was no quality assessment of the included studies, and the review excluded studies with participants on pharmacologic treatment for diabetes mellitus type 2, cardiovascular disease or smoking [19].

In summary, the literature on the effects of various exercise modalities in overweight and obese individuals is unclear. It is also not known whether concurrent caloric restrictions affect the effects of exercise modalities. The research questions the proposed systematic review and network meta-analysis will address, therefore, are the following:

1. Is one or more types of exercise more effective than others for overweight and obese adults in terms of body composition and metabolic health outcomes?
2. Do the effects of exercise modalities on body composition and metabolic health depend on caloric restriction?

## Methods/design

In line with current recommendations, the objective of this article is to describe the protocol for this systematic review and meta-analysis according to the preferred reporting items for systematic reviews and meta-analysis protocols (Additional file 1: PRISMA-P 2015 Guidelines) [17]. The protocol is registered in Prospero, the international prospective register of systematic reviews: CRD42019103371.

### Eligibility criteria

Studies will be selected according to the criteria described as follows.

#### Study design

We will include individual and cluster randomised controlled trials (RCTs). We will exclude studies using all other types of study designs.

#### Participants

We will include studies on overweight (body mass index (BMI) ≥ 25 kg/m^2^) and obese (BMI ≥ 30 kg/m^2^) human adults (18 years or older). Studies recruiting both adults and children will be included if data are reported separately for adults. We will exclude studies including individuals with severe comorbidities (e.g. cancer and stroke) and pregnant women. Furthermore, we will exclude studies designed to assess physical exercise for individuals who have undergone bariatric surgery and studies evaluating the effects of pharmacological treatments of obesity.

#### Interventions and comparators

The studies of interest will be interventions consisting of different types of exercise: aerobic exercise, resistance training, combined aerobic/resistance training and other types of training (e.g. flexibility training and body–mind training). Interventions must have a minimum duration of 4 weeks and should be at least partly supervised by a training instructor. The comparative arm can be any of the aforementioned exercise types, usual care or advice/encouragement about physical activity.

#### Outcomes

The primary outcomes are fat percentage and BMI. The secondary outcomes are body weight, body fat, lean body mass, waist and hip circumference and metabolic risk factors: HDL, LDL, total cholesterol, triglycerides, HOMA-IR, fasting glucose and HbA1C. Maximal/peak oxygen uptake is considered an additional outcome measure.

#### Follow-up times

We anticipate that the study authors will report response rates at various time points. We will subdivide the timing of outcome assessment accordingly:

1. Short-term effects: measured at the end of the intervention. This will be the primary time point reported.
2. Sustained effects: measured at 12-month follow-up after the intervention or the time point closest to 12 months (±2 months), if adequate data are available.

#### Setting

There will be no restrictions on the setting.

#### Language

We will only include studies published in English.

### Information sources

A comprehensive computerised search for relevant studies will be performed in the following bibliographic databases: PubMed, Embase (via Ovid) and CENTRAL (through the Cochrane Library). We will search for studies published from 2005 to the present. The Cochrane review by Shaw et al. [12], published in 2006, contains a literature search through 2005. Their search strategy is similar to ours, so we will search for studies published since 2005. We will also include older studies included in the systematic review by Shaw et al. In addition, we will inspect the reference lists of included studies and other relevant systematic reviews for relevant studies.

### Search strategies

A research librarian with expertise in systematic review search strategies will help develop the search strategies based on the following domains: overweight/obese and exercise combined with study design. A draft for the PubMed search strategy is available online (Additional file 2). Studies identified in the search will be uploaded to Endnote X9.

### Study selection

The reviewers will independently screen relevant records through a two-stage process. In the first stage, titles and abstracts will be screened, with the reviewers blinded to each other’s selections. Two reviewers will screen each title and abstract, and any disagreements will be discussed with and resolved by a third reviewer. Studies considered not to be relevant will be excluded. For the remaining studies, full-text articles will be obtained. Studies with uncertain relevance to the review will also be further considered in the second stage.

In the second stage, the reviewers will make the final selection based on screening of the full-text articles against the eligibility criteria. As in the first stage, the reviewers will be blinded to each other’s selections, and a third reviewer will be involved when disagreements occur. The reasons for excluding studies will be recorded in a flow chart to ensure the transparency of the process.

### Data extraction

Two reviewers will independently extract data from the included articles. Disagreements will be resolved by discussion with a third independent reviewer. To reduce potential errors, a pre-piloted form will be used to extract data (Additional file 3). We will extract the following data from the included studies, when available:

1. General information: authors, country, year of publication, study design and sample source.
2. Participants’ characteristics: age, gender, BMI and ethnicity.
3. Study characteristics: sample size and intervention content and duration.
4. Outcomes at the following time points: baseline, any intermediate time points, end of the intervention and end of follow-up data or change scores on the specified outcomes. Time points for measurements and the types of measurement methods (objective/subjective) will also be registered.

### Risk of bias assessment

Risk of bias will be assessed using the Cochrane risk of bias tool as outlined in *Cochrane Handbook for Systematic Reviews of Interventions* [20, 21], which recommends reporting on the following: (1) selection bias: sequence generation and allocation concealment, (2) performance bias: blinding of participants and personnel and other potential threats to validity, (3) detection bias: blinding of outcome assessment and other potential threats to validity, (4) attrition bias: incomplete outcome data and (5) reporting bias: selective outcome reporting. Each potential source for risk of bias will be assessed as high, low or unclear, and the ratings and the justifications will be presented in a table. Two independent reviewers will assess each study, and disagreements will be discussed with and resolved by a third independent author when necessary.

### Data analyses

The results of the data extraction and risk of bias assessment will be summarised in tables. It is unlikely that all the exercise modalities will be compared directly, so we will perform network meta-analysis if the assumptions are met (between-study homogeneity, transitivity and coherence across treatment comparisons) [22]. Network meta-analysis combines both the direct and the indirect evidence from different studies. The main analysis will be restricted to studies with low risk of bias. We will perform network meta-regression and subgroup analyses for gender, BMI (overweight and obese), intervention duration and amount of exercise in the interventions.

In addition, the following pair-wise comparisons are planned: endurance training vs resistance training; endurance training vs combined resistance and endurance training; and resistance training vs combined resistance and endurance training. The analyses will be stratified according to whether the participants have restricted caloric intake. If there are sufficient homogenous studies including body–mind training, they will also be compared to endurance training and strength training. We will perform meta-regression and subgroup analyses for the same variables as described in the network meta-analyses.

For continuous data, we will use the mean difference with 95% confidence intervals or the standardised mean difference with 95% confidence intervals if studies measure the same outcomes using different measurement scales. We will use change-from-baseline data or final value scores if change data are not available. We will perform sensitivity analyses stratified according to the risk of bias and analyses including all studies.

In the case of missing data, we will attempt to contact the authors to obtain the relevant missing data. We will also do so when the authors only present effect estimates for measures other than the mean values. All the missing data will be recorded. Small-study bias, such as publication bias, will be examined with the Eggers test and contour-enhanced funnel plots [23]. Heterogeneity will be assessed by graphically inspecting the confidence intervals for the individual studies, along with chi-squared test and the *I*^2^ [24]. The analyses will be performed as outlined in the *Cochrane Handbook for Systematic Reviews of Interventions* [21].

### Assessment of the strength of the evidence

The quality of the evidence for the main outcomes (fat percentage and BMI) will be judged using the Grading of Recommendations Assessment, Development and Evaluation (GRADE) working group methodology. The following factors will be assessed: risk of bias, consistency (heterogeneity or variability in the results), directness (direct or indirect comparison of interventions and differences in populations, interventions and comparators), precision and publication bias [25]. Other domains considered to be appropriate will also be included. As described in the GRADE approach, the quality will be judged as high (further research is very unlikely to change confidence in the estimated effect), moderate (further research is likely to have significant impacts on confidence in the estimated effect and may change the estimate), low (further research is very likely to have significant impacts on confidence in the estimated effect and is likely to change the estimate) or very low (any estimates of effect are very uncertain) [25, 26].

## Discussion

Obesity is a growing worldwide epidemic, and effective interventions are needed. Physical exercise is recognised as an important component in weight reduction and weight loss maintenance. We expect that in this systematic review, we will be able to identify whether certain exercise modalities with and without caloric restriction are more effective than others at improving body composition and metabolic health outcomes in overweight and obese adults.

The major strengths of this systematic review are the detailed protocol and search strategy developed with the help of an experienced research librarian. Prior reviews have generated unclear results on whether certain exercise modalities should be preferred. In addition, few systematic reviews have assessed the quality of studies in this area. In this review, we plan to conduct risk of bias assessment and perform network meta-analysis on only studies with low risk of bias. A potential problem is a loss of precision when excluding studies with high and unclear risk of bias. However, we contend that reduced bias is more important than precision. Nevertheless, we will perform sensitivity analyses stratified according to risk of bias and analyses including all studies. Another strength of this systematic review is the plan to present summary assessments using the GRADE approach to rate the quality of evidence, which offers transparent reporting that might help clinicians, patients and policy makers interpret the results. A limitation of this systematic review is the inclusion of only studies published in English, which might introduce bias. However, according to the *Cochrane Handbook for Systematic Reviews for Interventions* [21], the extent and effects of language bias might be diminished by a recent shift towards more studies published in English.

## Additional files

Additional file 1: PRISMA-P 2015 checklist. (DOCX 29 kb)

Additional file 2: Draft for the PubMed search strategy. (DOCX 12 kb)

Additional file 3: Data extraction form. (DOCX 23 kb)

## Abbreviations

BMI: Body mass index
GRADE: Grading of Recommendations Assessment, Development and Evaluation
