# Supplementary material for: What should be the preferred exercise modality for overweight and obese individuals? Protocol for a systematic review and network meta-analysis
Source: Syst Rev. 2019 Feb 4;8:41. doi: 10.1186/s13643-019-0964-1 (PMC6360785; doi:10.1186/s13643-019-0964-1)
Supplement: Supplementary file 2 — Draft for the PubMed search strategy. (DOCX 12 kb) [file 13643_2019_964_MOESM2_ESM.docx]

**Online supplementary file 1**

**Draft for the PubMed search strategy**

(Overweight [mesh] OR weight loss [mesh:noexp] OR obese [tiab] OR obesity [tiab] OR overweight [tiab] OR "weight reduction" [tiab] OR "weight loss" [tiab] OR overfat [tiab]) AND (Exercise [mesh] OR Exercise therapy [mesh] OR exercise* [tiab] OR training [tiab] OR (physical [tiab] AND conditioning [tiab]) OR "physical activity" [tiab]) AND English [la] AND 2005 [pdat]:3000 [pdat] AND (randomized controlled trial [pt] OR randomi* [tiab])
