# Supplementary material for: What should be the preferred exercise modality for overweight and obese individuals? Protocol for a systematic review and network meta-analysis
Source: Syst Rev. 2019 Feb 4;8:41. doi: 10.1186/s13643-019-0964-1 (PMC6360785; doi:10.1186/s13643-019-0964-1)
Supplement: Supplementary file 3 — Data extraction form. (DOCX 23 kb) [file 13643_2019_964_MOESM3_ESM.docx]

**Online supplementary file 2**

**Data extraction form**

| **Study details** | | | | | |
| --- | --- | --- | --- | --- | --- |
| Author |  | | | | |
| Publication year |  | | | | |
| Title |  | | | | |
| Country of study |  | | | | |
| Study design |  | | | | |
|  |  | | | | |
| **Inclusion criteria in the study** |  | | | | |
| Age |  | BMI: | | | |
| Gender |  | | | | |
| Other (e.g. Diagnoses) |  | | | | |
|  |  | | | | |
|  |  | | | | |
| **Interventions** | | | | | |
| Diet included | Yes/no | | | | |
|  |  | | | | |
| Intervention arms (short title) and number of participants | Full name | | | Short name | Number of participants in each arm |
| 1 |  | | |  |  |
| 2 |  | | |  |  |
| 3 |  | | |  |  |
| 4 |  | | |  |  |
|  |  | | |  |  |
| Content in  intervention arms | 1. | | | | |
|  | 2. | | | | |
|  | 3. | | | | |
|  | 4. | | | | |
| Intervention length | Weeks: | | Times per week: | | |
| Supervised exercise | Yes: | No: | Partly: | | |
| Follow-up time-points |  | | | | |
| How are missing values handled |  | | | | |
| Analyses | Intention to treat  Complete case  Mixed model  Multiple imputation | | | | |

| **Outcomes/Results** | | | | | | | | | | | | |
| --- | --- | --- | --- | --- | --- | --- | --- | --- | --- | --- | --- | --- |
| Participants | | | | | | | | | | | | |
| N |  | | | | Age mean: | | |  | | | |  |
| Gender | Men N: | | | | Men | | | Women N: | | | | Women %: |
| BMI mean |  | | | |  | | |  | | | |  |
| Ethnicity | N/R: | | | | Asian | | | Other | | | |  |
|  | | | | | | | | | | | | |
| Time points for evaluation | |  | | | | | | | | | | |
| Results presented as | | Baseline:  Follow-up:  Change scores  Ancova: | | | | | | | | | | |
|  | | | | | | | | | | | | |
| Type of variation measurement | | SD  SE  SEM  CI | | | | | | | | | | |
|  | | Time point: | | Time point: | | | Time point: | | | Change score: | |  |
|  | | mean | SD/SE/  SEM/CI | mean | | SD/SE/  SEM/CI | mean | | SD/SE/  SEM/CI | Mean difference | SD/SE/  SEM/CI | Scale used (e.g. kg, percent, cm) |
| ***Anthropometrics*** | |  |  |  | |  |  | |  |  |  |  |
| Fatpercent | |  |  |  | |  |  | |  |  |  |  |
| Body fat | |  |  |  | |  |  | |  |  |  |  |
| Weight | |  |  |  | |  |  | |  |  |  |  |
| BMI | |  |  |  | |  |  | |  |  |  |  |
| Lean body mass | |  |  |  | |  |  | |  |  |  |  |
| Waist circumference | |  |  |  | |  |  | |  |  |  |  |
| Hip circumference | |  |  |  | |  |  | |  |  |  |  |
| Waist to hip ratio | |  |  |  | |  |  | |  |  |  |  |
| ***Metabolic*** | |  |  |  | |  |  | |  |  |  |  |
| HDL | |  |  |  | |  |  | |  |  |  |  |
| LDL | |  |  |  | |  |  | |  |  |  |  |
| Total-cholesterol | |  |  |  | |  |  | |  |  |  |  |
| Triglycerides | |  |  |  | |  |  | |  |  |  |  |
| HOMA-IR | |  |  |  | |  |  | |  |  |  |  |
| Fasting glucose | |  |  |  | |  |  | |  |  |  |  |
| HbA1C | |  |  |  | |  |  | |  |  |  |  |
|  | |  |  |  | |  |  | |  |  |  |  |
| ***Other*** | |  |  |  | |  |  | |  |  |  |  |
| Maximal/peak oxygen uptake | |  |  |  | |  |  | |  |  |  |  |
|  | |  |  |  | |  |  | |  |  |  |  |
| Type of measurement methods | |  | | | | | | | | | | |
| Comments to measurements | |  | | | | | | | | | | |

| **Quality assessment** | | |
| --- | --- | --- |
|  | **Judgement:**  **Low risk/high risk/unclear risk** | **Support for judgment**  **Quote and comment** |
| Random sequence generation (selection bias) |  |  |
| Allocation concealment (selection bias) |  |  |
| Blinding of participants and personnel (performance bias) |  |  |
| Blinding of outcome assessment (detection bias) (should be assessed for each main outcome) | |  |
| Body composition outcomes |  |  |
| Metabolic risk factors |  |  |
|  |  |  |
| Incomplete outcome data addressed (attrition bias)  (missing data, only per protocol) |  |  |
| Selective reporting (reporting bias) |  |  |
| Other bias.  Bias due to problems not covered elsewhere in the table |  |  |
